# Supplementary material for: Knowledge, attitude, and practice of pharmacy professionals on generic medicines in Eastern Ethiopia: A cross-sectional study
Source: PLoS One. 2020 Jul 13;15(7):e0235205. doi: 10.1371/journal.pone.0235205 (PMC7357759; doi:10.1371/journal.pone.0235205)
Supplement: S1 File — (DOCX) [file pone.0235205.s001.docx]

**Consent Information Sheet**

My name is __________________________. I am here to conduct a research on **‘Knowledge, Attitude and Practice of Pharmacy Professionals towards Generic Medicines, Eastern Ethiopia** The research project is approved by the Research Ethics committee of School of Pharmacy, Haramaya University. Your participation is purely based on your willingness and you have the right to choose not to take part in this study. If you choose to take part, you have the right to stop at any time.

If you agree to participate in the study, you will be asked to answer some questions about yourself and your knowledge, attitude and practice towards generic medicines as well as perceived barriers in dispensing of generic medicine. It took only 30 minute to fill the query. The information that you provide will be kept confidential by using only code numbers and locking the data.

Based on the understanding of the information I gave you, are you willing to participate in this study?

1. Yes
2. No

**Questionnaire**

**PART** **I - Socio demographic characteristic of the study participants.**

Read each question carefully and put your answer in box lie ahead of each choice

1. Age _______Years

2. Sex A) Male [ ] B) Female [ ]

3. What is your qualification?

A) Diploma [ ] B) Degree [ ] C) Msc

4. What is your employment status?

A) Pharmacy/drug store owner [ ] B) employee [ ] C) Others [ ]

5. What is your education institution?

A) Private institution [ ] B) government institution [ ]

6. How long is your work experience?

A) Less than 5 years [ ] B) 5 and above years

7. How many prescription you filled per day

A) Up to 10 [ ] B) 11-20 [ ] C) 21-30 [ ] D)>30 [ ]

8. How much is your monthly salary

A) Less than 1499 birr [ ] B) 1499-30000 birr [ ]

C) Greater than 3000 birr [ ]

**Part2- Knowledge, Attitude and practice of community pharmacist’s medicine and also their view towards generic medicines**

Read each question carefully and put your choice in the table box while SD=strongly disagree D= disagree NE=neutral A= agree SA=strongly agree stands for.

| **Variables** | SD | D | NE | A | SA |
| --- | --- | --- | --- | --- | --- |
| **knowledge** | | | | | |
| A generic medicine is bioequivalent to brand medicine |  |  |  |  |  |
| generic medicine must contain the same amount of active ingredients as the brand medicine |  |  |  |  |  |
| A generic medicine must be in the same dosage form as the brand medicine |  |  |  |  |  |
| Generic medicines are cheaper than brand name medicines |  |  |  |  |  |
| Wider use of generic medicines in Ethiopia helps in decreasing the health care expenditure of government |  |  |  |  |  |
| Community pharmacists in Ethiopia have the right to perform generic substitution |  |  |  |  |  |
| Substitution of medicines with narrow therapeutic index is inappropriate |  |  |  |  |  |
| In Ethiopia generic medicines are approved by FMHACA just like brand medicines |  |  |  |  |  |
| **Attitude** | | | | | |
| Generic medicines are less effective compared to brand name medicines |  |  |  |  |  |
| Brand name medicines are of higher quality compared to generic drugs |  |  |  |  |  |
| Generic drugs produce more side effects than brand name medicines |  |  |  |  |  |
| Generic medicines take longer time to give response |  |  |  |  |  |
| I support generic substitution for brand name medicines in all cases where s generic is available |  |  |  |  |  |
| Price difference between generic and brand medicine is often so great that I feel I must dispense prescriptions with generic substitution especially for people who do not afford. |  |  |  |  |  |
| Patients should be given enough explanations about the reasons for choosing generic medicines |  |  |  |  |  |
| The intensity of promotional activities by promoters plays an important role in dispensing brand medicines |  |  |  |  |  |
| Community pharmacists should be allowed to perform generic substitutions without consulting prescribing physicians |  |  |  |  |  |
| **View of pharmacy professional toward locally manufactured drugs** | | | | | |
| \| Locally manufactured generics are equal in their quality compared to the imported generics \| \| --- \| |  |  |  |  |  |
| Locally manufactured generics are equal in their safety and efficacy compared to the imported generic |  |  |  |  |  |
| Manufacturers of local generic products have a reliable logistic and supply system |  |  |  |  |  |
| I prefer to stock and dispense locally manufactured generics because the companies provide good bonus scheme compared to suppliers importing them |  |  |  |  |  |
| Credibility of the generic manufactures/suppliers is my concern when stocking medicines in my pharmacy |  |  |  |  |  |
| I will only stock locally manufactured product which is well advertised through medical representatives and medicine related references. |  |  |  |  |  |
| Imported generics need to pass more stringent approval process compared with locally manufactured ones. |  |  |  |  |  |
| Locally manufactured generics are cheaper compared to imported generics. |  |  |  |  |  |
| Drug Regulatory Authorities need to convince pharmacists that registered locally manufactured generics are of high quality and standards. |  |  |  |  |  |

| **Possible influencing factors related to selection and dispensing of generic medicines among the community pharmacists**. | Not important | Neutral | Important |
| --- | --- | --- | --- |
| Lack of belief in generic medicines |  |  |  |
| Availability of policies, laws &regulations |  |  |  |
| Affordability to the customer |  |  |  |
| Lacking options |  |  |  |
| Consumer preference/ demand |  |  |  |
| Cost effectiveness of generic medicines |  |  |  |
| Substitution agreement with prescriber |  |  |  |

**በጥናቱ ላይ ለመሳተፍ ፍቃደኝነት መጠየቂያ**

ስሜ ………………………….. ሲሆን በአሁኑ ሰዓት የፋርማሲ ባለሙያዎች በጄኔሪክ ሜድሰን ላይ ያላቸውን እውቀት፣አመለካከትና ትግበራ ላይ ያተኮረ ጥናት በመስራት ላይ ነኝ፡፡ ይህ ጥናታዊ ፅሁፍ በሀረሚያ ዩኒቨርስቲ የጥናትና ምርምር ኮሚቴ የፀደቀ ሲሆን የእናንተ ተሳትፎ በፍላጐት ላይ የተመሰረተ ነው፡፡ በጥናት ላይ መሳተፍ ካልፈለጉ ያለመሳተፍ ሙሉ መብትዎ የተጠበቀ ነው በተጨማሪም በጥናቱ ላይ በመሳተፍ ላይ እያሉ በመሀል ለማቆም ከፈለጉ አሁንም ሙሉ መብትዎ እንደተጠበቀ ነው፡፡

በጥናቱ ላይ በሚሳተፍበት ወቅት የተለያዩ ጥያቄዎች ሊጠየቁ ይችላሉ፡፡ ጥናቱ ላይ ለመሳተፍ ከተስማሙ ጥናቱ የሚያማክላቸው ጥያቄዎች መመለስ ይጠበቅቦታል፡፡ ጥያቄዎቹም በጄኔሪክ ሜድሰን ላይ ያሎትን እውቀት፣አመለካከትና ትግበራ ያማከለ ሲሆን ጄኔሪክ ሜድሰን ለማከፋፈል ሊያግድ ስለሚችል እንከኖችም ይጠይቃል፡፡ መጠይቁ 30 ደቂቃ የሚፈጀ ሲሆን ከእርሶ የተወሰደው መረጃ ማንነቶን አይለይም፡፡

ከላይ በተላለፈሎት መረጃ መሰረት በጥናቱ ላይ ለመሳትፍ ፍቃደኛ ኖት

1. አዎ 2. አይደለሁም

**መጠይቅ**

**ክፍል 1፦ የጥናቱ ተሳታፊዎች መሀበራዊ እና አኗናር መረጃ**

| 1. እድሜ _____ |
| --- |
| 1. ፆታ 1 ወንድ 2 ሴት |
| 1. የትምህርት ደረጃ 1.ዲፕሎማ 2.ዲግሪ |
| 1. የቅጥር ሁኔታ   1.የፋርማሲ/የመድሀኒት መደብር ባለቤት/ 2.ተቀጣሪ |
| 1. ከፍተኛ የትምህርት ደረጃዎን የተማሩት የት ነው? 1. የግል ተቋም 2. የመንግስት ተቋም |
| 1. የስራ ልምዶት ምን ያህል ነው?   1.ከአምት ዓመት ያንሳል 2. አምስት አመትና ከዛ በላይ ነው |
| 1. በቀን ምን ያህል የመድሃኒት ማዘዣ ትሞላለለህ?   1.እስከ 10 3. ከ21-30  2.ከ11-20 4. >30 |
| 1. በወር የሚከፈሎት ደመወዝ ምን ያህል ነው ?   1. 1499 ብር ያንሳል  2. ከ1499-3000 ብር  3. ከ3000 ብር ይበልጣል |

**ክፍል 2፡-** ከጀነሪክ ስም የሚሸጥ መድሀኒትና ላይ ያላችሁ እውቀት፣ አመለካከት ትግበራ

**ከዚህ በመቀጠል ያሉትን ጥያቄዎች በጥንቃቄ በማንበብ የመረጡትን ምርጫ በተዘጋጀው ሳጥን ውስጥ የ “X” ምልክት ያስቀምጡ**

|  | በጣም አልስማማም | አልስማማም | መካከለኛ | እስማማለሁ | በጣም እስማማለሁ |
| --- | --- | --- | --- | --- | --- |
| **በጄኔሪክ ሜድስን ላይ ያሎት እውቀት** | | | | | |
| ጄኔሪክ መድሀኒቶች ከብራንድ መድሀኒቶች ጋር ባዮ ኢኪውቫለንት ናቸው |  |  |  |  |  |
| ጄኔሪክ መድሀኒቶች ኩብራንድ መድሃኒቶች ጋር ተመሳሳይ የሆነ አክቲቭ ኢንግሪዲየንት ሊኖራቸው ይገባል፡፡ |  |  |  |  |  |
| ጄኔሪክ መድሀኒቶች ከብራንድ መድሀኒቶች ጋር ተመሳሳይ ዶዜጅ ፎርም መሆን አለባቸው |  |  |  |  |  |
| ጄኔሪክ መድሀኒቶች ከብራንድ መድሀኒቶች አንፃር ዋጋቸው ይቀንሳል፡፡ |  |  |  |  |  |
| ጄኔሪክ መድሀኒቶችን በስፋት መጠቀም መንግስት ለጤና ጥበቃ የሚያወጣውን ወጪ ለመቀነስ ይረዳል |  |  |  |  |  |
| በኢትዮጵያ ውስጥ ያሉ የመንደር ፋርማሲ ባለሙያዎች ጄኔሪክ መድሀኒቶችን ማተካካት መብት አላቸው፡፡ |  |  |  |  |  |
| መድሀኒቶችን ናሮ ቴራፑውቲክስ ኢንዴክስ ባላቸው ሌላ መድሀኒቶች መተካት ተገቢ አይደለም |  |  |  |  |  |
| በኢትዮጵያ ውስጥ ጄኔሪክ መድሀኒቶች እንደ ብራክድ መድሀኒቶት ለጥቅም የሚውሉት በFMHACA ሲፀድቅላቸው ነው፡፡ |  |  |  |  |  |
| **በጄኔሪክ ሜድስን ላይ ያሎት አመለካከት** | | | | | |
| ጄኔሪክ መድሀኒቶች ፍቱንነታቸው ከብራንድ መድሀኒቶች አንፃር ያንሳል |  |  |  |  |  |
| ብራንድ ስም ያላቸው መድሀኒቶች ከጄኔሪክ አንፃር ከፍ ያለ ጥራት አላቸው |  |  |  |  |  |
| ጄኔሪክ መድሀኒቶች ከብራንድ መድሀኒቶች አንፃር ተጓዳኛ ጉዳታቸው ይጨምራል |  |  |  |  |  |
| ጄኔሪክ መድሀኒቶች ለማዳን ረዘም ያለ ጊዜ ይወስዳሉ |  |  |  |  |  |
| በማንኛውም ጊዜ ጄኔሪክ መድሀኒቶች ሲኖሩ ጄኔሪክ መድሀኒቶችን በብራንድ መድሀኒቶች መተካቱን እደግፋለሁ |  |  |  |  |  |
| በጄኔሪክ መድሀኒት እና በብራንድ መድሀኒት መካከል የዋጋ ልዩነት ስላለ መድሃኒቱን ማግኘት ለማይችሉ ታካሚዎች ጄኔሪክ መድሀኒቶች መተካት እንዳለብኝ ይሰማኛል |  |  |  |  |  |
| ህመምተኞች ስለ ጄኔሪክ መድሀኒት በቂ የሆነ ማብራሪያ ሊሰጣቸው ይገባል |  |  |  |  |  |
| የመድሀኒት አስተዋዋቂዎች የማስተዋዋቅ ብቃት ብራንድ መድሀኒቶችን ተደራሽ ለማድረግ ጠቃሚ ሚና ይጫወታል |  |  |  |  |  |
| የመንደር ፋርማሲዎች የዶክተሮች ምክር ሳያስፈልጋቸው ጄኔሪክ መድሀኒቶችን የመተካት መብት ሊኖራቸው ይገባል |  |  |  |  |  |
| **የፋርማሲ ባለሙያዎች ሀገር ውሰጥ ለሚመረቱ መድሀኒቶች ያላቸው እይታ** | | | | | |
| በሀገር ውስጥ የሚመረቱ ጄኔሪክ መድሀኒቶች ከውጭ ሀገር ከሚመረቱት ጋር እኩል ጥራት አላቸው |  |  |  |  |  |
| በሀገር ውስጥ የሚመረቱን ጄኔሪክ መድሀኒት ከውጭ ሀገር ከሚገቡት ጋር እኩል የሆነ ደህንነት እና ፍቱንነት አላቸው |  |  |  |  |  |
| የሀገር ውስጥ ጄኔሪክ ምርቶች ለመታመን የሚችል ሎጀስቲክ እና ስፕላይ ሲስተም አላቸው፡፡ |  |  |  |  |  |
| ሀገር ውስጥ የሚመረቱ መድሀኒቶችን መግዛትና ለህመምተኞች ማከፋፈል ከውጭ ከማስገባት ይልቅ ተመራጭ ናቸው ምክንያቱም የሀገር ውሰጥ አመራቾች ተጨማሪ/ / መድሀኒት ስለሚሱጡ |  |  |  |  |  |
| ለፋርማሲ መድሀኒት በማስገባበት ወቅት የጄኔሪክ መድሀኒት አምራቾች ተአሚኒነትን ማረጋገጥ የመጀመሪያ ስራዬ ነው |  |  |  |  |  |
| በሀገር ውሰጥ የሚመረቱ መድሃኒቶች የምገዛው በጥሩ ሁኔታ በጤና ባለሙያ ተወካዮች እና የመድሃኒት መረጃ ሰጪዎች በአግባቡ ከተዋወቁ ነው |  |  |  |  |  |
| ከውጪ የሚገቡት መድሀኒቶች ሀገር ውስጥ የሚመረቱት አንፃር ጠበቅ የአፀዳደቅ ስርዓት ሊኖራቸው ይገባል |  |  |  |  |  |
| በሀገር ውስጥ የሚመረቱ ጄኔሪክ መድሀኒቶች ከውጪ ከሚገቡት አንፃር ዋጋቸው ቅናሽ ነው |  |  |  |  |  |
| የመድሃኒት ቁጥጥር ባለስልጣናት ለፋርማሲ ባለሙያዎች በሀገር ውስጥ ስለሚመረቱን ጄኔሪክ መድሀኒቶች ጥራትና የተወዳዳሪነት ደረጃ ማሳመን መቻል አለባቸው |  |  |  |  |  |

**ጄኔሪክ መድሃኒቶችን ለመምረጥና ለታማሚዎች ተደራሽ ለማድረግ ሊያግዱ የሚችሉ እንከኖች**

|  | ኣስፈላጊ ኣይደለም | ታቅቦ | ኣስፈላጊ ነው |
| --- | --- | --- | --- |
| በጄኔሪክመድሀኒቶች ላይ እምነት አለመኖር |  |  |  |
| ፖሊስ፣ህግን ደንብ መኖር |  |  |  |
| ለተጠቃሚ ተደራሽ መሆን |  |  |  |
| የአማራጭ መድሀኒቶች እጥረት መኖር |  |  |  |
| የደንበኞች ምርጫ |  |  |  |
| የጄኔሪክ መድሃኒቶች ዋጋቸው ጥሩ መሆን |  |  |  |
| ከደብተሮች /ከመድሀኒት አዛዣች ጋር/ መድሀኒቶችን ለመተካካት መስማማት |  |  |  |
