# Supplementary material for: Knowledge, attitude, and practice of pharmacy professionals on generic medicines in Eastern Ethiopia: A cross-sectional study
Source: PLoS One. 2020 Jul 13;15(7):e0235205. doi: 10.1371/journal.pone.0235205 (PMC7357759; doi:10.1371/journal.pone.0235205)
Supplement: Attachment — Submitted filename: Response to reviewers.docx [file pone.0235205.s003.docx]

**Response to editor**

Dear Editor-in-chief,

We are thankful to both editor and reviewer for their effort and constructive comments on our manuscript. We have executed their comments and recommendations and wish to submit a revised version of the manuscript for further consideration in your journal. Changes in the initial version of the manuscript are either highlighted for added sentences or strikethrough for deleted sentences in the revised version. Below, we also provide answer for each point rose by editor and reviewer, elucidating how we have addressed each of comments. We look forward to the result of your assessment.

Yours sincerely,

On behalf of the co-authors

Ammas Siraj, B Pharm

**Editor(s)’ decision and comments**

Thank you for submitting your manuscript to PLOS ONE. After careful consideration, we feel that it has merit but does not fully meet PLOS ONE’s publication criteria as it currently stands. Therefore, we invite you to submit a revised version of the manuscript that addresses the points raised during the review process.

Our answer: thank you for your consideration of its merit and offer.

If you would like to make changes to your financial disclosure, please include your updated statement in your cover letter.

Our answer: thank you. No change in financial disclosure

To enhance the reproducibility of your results, we recommend that if applicable you deposit your laboratory protocols in [protocols.io](http://protocols.io/), where a protocol can be assigned its own identifier (DOI) such that it can be cited independently in the future. For instructions see: <http://journals.plos.org/plosone/s/submission-guidelines#loc-laboratory-protocols>

Our answer: thank you. It is not applicable

**Response to editor**

**1.** Please ensure that your manuscript meets PLOS ONE's style requirements, including those for file naming. The PLOS ONE style templates can be found at <http://www.plosone.org/attachments/PLOSOne_formatting_sample_main_body.pdf> and <http://www.plosone.org/attachments/PLOSOne_formatting_sample_title_authors_affiliations.pdf>

Our Answer: Thank you for this comment. The revised manuscript version is formatted in strict adherence to PLOS ONE’s formatting guideline, which obtained from above URL address, including file naming, font size for each section, spacing and other required format.

**2.** Please provide additional details regarding participant consent. In the ethics statement in the Methods and online submission information, please ensure that you have specified whether consent was written or verbal/oral. If consent was verbal/oral, please specify: 1) whether the ethics committee approved the verbal/oral consent procedure, 2) why written consent could not be obtained, and 3) how verbal/oral consent was recorded. If your study included minors, please state whether you obtained consent from parents or guardians in these cases.

Our answer: Thank you for rising this point. Since the study participants are expected to read and write, written informed consent was obtained from all subjects before proceeding to query, which was approved by ethical review committee. During the informed consent process, study participants were assured that data collected would be used only for stated purposes and would not be disclosed or released to others without the consent of the participants. All study participants was adults, pharmacy professionals.

**3.** Please include copies of the survey questions or questionnaires used in the study, in both the original language and English, as Supporting Information, or include a citation if they have been published previously.

Our answer: Thank you for this comment. The questionnaire used in this study is not published anywhere, but the literature we derived it from were adequately cited and it is provided as supplementary material both in local language and English upon submission.

**4**. We noticed you have some minor occurrence of overlapping text with the following previous publication(s), which needs to be addressed:
<https://www.jbclinpharm.org/articles/assessment-of-knowledge-attitude-and-practice-of-pharmacy-professionals-toward-generic-medicines-northern-ethiopia-mekelle-a-cross-3923.html>. In your revision ensure you cite all your sources (including your own works), and quote or rephrase any duplicated text outside the Methods section. Further consideration is dependent on these concerns being addressed.

Our answer: thank you for rising this point. We have checked the manuscript for any possible overlapping text to different source including the one you have indicated above and the obtained overlapping text were rephrased and synthesized using authors’ specific language

**5.** We suggest you thoroughly copyedit your manuscript for language usage, spelling, and grammar. If you do not know anyone who can help you do this, you may wish to consider employing a professional scientific editing service.

Our answer: thank you for this comment. After copyediting the manuscript thoroughly, we have tried to address all typographical and grammatical errors in the manuscript. The grammatical and typographical errors within manuscript was addressed in collaboration with my colleagues

**6.** Your ethics statement must appear in the Methods section of your manuscript. If your ethics statement is written in any section besides the Methods, please move it to the Methods section and delete it from any other section. Please also ensure that your ethics statement is included in your manuscript, as the ethics section of your online submission will not be published alongside your manuscript.

Our answer: we have moved the ethics statement from declaration section to method section of the manuscript.

**7.** We note you have included a table to which you do not refer in the text of your manuscript. Please ensure that you refer to Table 2 in your text; if accepted, production will need this reference to link the reader to the Table.

Our answer: we have included citation of table2 in the text prior to the table as per your recommendation.

**Reviewers comment and decision**

1. Is the manuscript technically sound, and do the data support the conclusions?

   The manuscript must describe a technically sound piece of scientific research with data that supports the conclusions. Experiments must have been conducted rigorously, with appropriate controls, replication, and sample sizes. The conclusions must be drawn appropriately based on the data presented. 

   Reviewer #1: Yes
2. Has the statistical analysis been performed appropriately and rigorously?

   Reviewer #1: Yes
3. Have the authors made all data underlying the findings in their manuscript fully available?

   The [PLOS Data policy](http://www.plosone.org/static/policies.action#sharing) requires authors to make all data underlying the findings described in their manuscript fully available without restriction, with rare exception (please refer to the Data Availability Statement in the manuscript PDF file). The data should be provided as part of the manuscript or its supporting information, or deposited to a public repository. For example, in addition to summary statistics, the data points behind means, medians and variance measures should be available. If there are restrictions on publicly sharing data—e.g. participant privacy or use of data from a third party—those must be specified.

   Reviewer #1: Yes
4. Is the manuscript presented in an intelligible fashion and written in standard English?

   PLOS ONE does not copyedit accepted manuscripts, so the language in submitted articles must be clear, correct, and unambiguous. Any typographical or grammatical errors should be corrected at revision, so please note any specific errors here.

**Response to reviewer**

Reviewer #1: This is an interesting study to uncover the knowledge, beliefs and attitudes among pharmacists in Ethiopia and other related professionals, about the value of generic drugs. The approach and methods are well described. The authors should describe the pharmacy practice system at the beginning, so that the results are put in the context of pharmacy practice and can be better interpreted.
In the discussion, there should also be a more thorough explanation of the 'so what' and the next steps.
In general, there are many grammatical and typographical errors that needs to be corrected, and the formatting should be improved

Our answer: thank you for your appreciation and offer. Pharmacy practice system is described at the beginning of manuscript including its relation with generic drug utilization and we have tried to put and interpret the result in context of pharmacy practice system. In discussion, we have included that what should be done per our each finding and at the end of discussion, future direction for studies that will be conducted on the area. After looking the manuscript thoroughly, we have tried to address all grammatical and typographical errors in manuscript and the revised version of manuscript is formatted in strict adherence to PLOS ONE’s formatting style including naming of file.
